# DGCR8/miR-106 Axis Enhances Radiosensitivity of Head and Neck Squamous Cell Carcinomas by Downregulating RUNX3

**DOI:** 10.3389/fmed.2020.582097

**Published:** 2020-12-15

**Authors:** Chunlin Zhang, Hangqi Chen, Zeyi Deng, Dan Long, Li Xu, Zhaohui Liu

**Affiliations:** ^1^Department of Otorhinolaryngology, Head and Neck Surgery, Affiliated Hospital of Zunyi Medical University, Zunyi, China; ^2^Department of Otorhinolaryngology, Head and Neck Surgery, Zhujiang Hospital, Southern Medical University, Guangzhou, China

**Keywords:** head and neck cancer, HPV, DGCCR8, miR-106a, RUNX3, radiotherapy

## Abstract

**Purpose:** Head and neck squamous cell carcinoma (HNSCC) is the sixth most prevalent malignant tumor worldwide, and the radiotherapy effect is strongly associated with human papillomavirus (HPV) infection. Therefore, the aim of our study was to analyze the mechanism of HPV E7 and its effects on radiosensitivity in HNSCC cells.

**Methods:** The mRNA expression of DiGeorge syndrome critical region gene 8 (DGCR8), has-miR-106a, and Runt-related transcription factor 3 (RUNX3) was examined by quantitative real-time PCR (RT-qPCR). The protein expression of DGCR8, E7, RUNX3, caspase-3/cleaved caspase-3, poly(ADP-ribose) polymerase (PARP)/cleaved PARP, and γH2AX was measured by Western blot. The expression level of DGCR8 was measured by immunofluorescence assay. Starbase database (http://starbase.sysu.edu.cn/) was used to analyze the correlation between has-miR-106a-5p and DGCR8. TargetScan database (http://www.targetscan.org/vert_72/) was adopted to calculate the prediction of binding sites. Radiosensitivity was evaluated through clone formation assays and Cell Counting Kit-8 (CCK-8) assays.

**Results:** In our study, we found that the mRNA and protein expression levels of HPV E7 and DGCR8 in HPV-positive HNSCC cells were higher than those in HPV-negative cells. The expression of DGCR8 was increased in FaDu and UM-SCC-4 with E7 overexpression, while the expression of DGCR8 was decreased in UM-SCC-47 and UPCI-SCC-090 with E7 silence. The miR-106a expression was increased after DGCR8 overexpression in FaDu and UM-SCC-4. However, the miR-106a expression was decreased in UM-SCC-47 and UPCI-SCC-090 with E7 silence. In radiation conditions, clone formation assays found that less clones formed in FaDu and UM-SCC-4 cells subsequent to silencing DGCR8 or miR-106a than that in the control group, and more clones were formed in UM-SCC-47 and UPCI-SCC-090 cells overexpressing DGCR8 or miR-106a than that in the control group. Luciferase reporter gene assays verified that miR-106a targeted the 3′ untranslated region (UTR) of RUNX3 mRNA. MiR-106a overexpression resulted in a decrease in RUNX3 expression, and miR-106a silence increased RUNX3 expression. Rescue experiments conducted with miR-106a inhibitor restored radiation resistance and reduced DNA damage in radiation condition.

**Conclusions:** Our study indicated that HPV E7 activated DGCR8/miR-106a/RUNX3 axis to enhance radiation sensitivity and provided directions for targeted therapeutic interventions.

## Background

Head and neck cell carcinoma is one of the most common malignancies, and its incidence rate ranks sixth among all cancers (1). Over 600,000 new cases are diagnosed as head and neck cell carcinoma every year worldwide, and the incidence is increasing year by year (2). Ten percent of the cases are in the oropharynx approximately. Head and neck squamous cell carcinoma (HNSCC) is the most common kind of head and neck cancer (3). Due to the lack of indicators for early diagnosis, HNSCC cannot be easily detected and ~60% of patients with HNSCC are already at an advanced stage at the time of treatment (4). Smoking and alcohol are the principal risk factors for HNSCC worldwide (5). Currently, surgery, radiotherapy, and chemotherapy make a great progress, but the overall survival rate of patients has not been improved. The 5-year survival rate is <50% (6).

Human papillomavirus (HPV) E6/E7 oncoproteins represent potentially ideal targets for therapeutics (7) based on a previous study, but the possible mechanism was not in-depth. Currently, it is believed that HPV infection is a relevant causative factor in the development of HNSCC. The expression of HPV E6/E7 causes tumorigenesis and degrades the expression products of the tumor suppressor genes p53 and retinoblastoma protein (pRb) (8). HPV E7 represses Rb activity and reversely activates multiple transcription factors related to the tumor process (9). HPV-negative and HPV-positive patients have different responses to treatment and prognosis (10, 11). It is shown that HPV E6/E7 enhances the sensitivity of radiotherapy in HNSCC.

Recently, some studies have found that HPV E7 affects miRNA expression profiles *via* DiGeorge syndrome critical region gene 8 (DGCR8) (12, 13). In the nucleus, miRNAs are transcribed into pri-miRNAs and are processed to pre-miRNAs by Drosha and DGCR8 (14). DGCR8 is a factor of the microprocessor complex and has been shown to be necessary for miRNA maturation (15). High-risk HPV does not have its own miRNA but alters miRNA level in host cells. The effects of HPV E6/E7 on the maintenance of cell invasion and proliferation or drug resistance and radiosensitivity through miRNAs have not been demonstrated clearly.

Runt-related transcription factor 3 (RUNX3) is a member of the runt domain-containing family of transcription factors (16–18). It is involved in the control of cellular proliferation and differentiation. Samarakkody et al. (19) and Tay et al. (20) demonstrated that RUNX3 participated in DNA damage repair, which radiation mainly caused. In addition, RUNX3 is an proto-oncogene in head and neck cancer (21). RUNX3 expression is a useful marker and therapeutic target to predict malignant behaviors and radiotherapy in HNSCC.

Similar to a previous study, we found that HPV-positive HNSCC cells were more sensitive to radiotherapy. In our study, we proved that HPV E7 could promote the content of DGCR8, a protein that affected miRNA maturation, the transcription of hsa-miR-106a, and thus disinhibited RUNX3 expression in HNSCC. Thereby, it can enhance the sensitivity of radiotherapy, which potentially provides directions for targeted therapeutic interventions.

## Methods

### Cell Cultures and Transfection

HPV-negative HNSCC cell lines FaDu, UM-SCC-4, and HPV-positive cell line UM-SCC-47, UPCI-SCC-090, were purchased from American Type Culture Collection (ATCC). FaDu and UM-SCC-47 were cultured in Dulbecco's modified Eagle's medium (DMEM) (Gibco, Carlsbad, CA) with 10% fetal bovine serum (FBS). UM-SCC-4 was cultured in DMEM/F12 medium (Gibco, Carlsbad, CA) with 10% FBS. UPCI-SCC-090 was cultured in Minimum Essential Medium (MEM) (Gibco, Carlsbad, CA) with 10% FBS. All cells were cultured in an incubator at 37°C with 5% CO_2_. Cells were allowed to acclimate for 24 h before any treatment in all experiments. SiRNAs were purchased from RiboBio (Guangzhou, China), and overexpression/luciferase reporter plasmids were purchased from GeneChem (Shanghai, China). Cells were transiently transfected with corresponding siRNAs or overexpression/luciferase reporter plasmids using Lipofectamine 3000 Transfection Reagent (Invitrogen, United States) according to the manufacturer's instructions. After transfection for 48 h, the cells were collected for further analysis.

### Western Blot

Cells were lysed with radioimmunoprecipitation assay (RIPA) buffer with phenylmethylsulfonyl fluoride (PMSF) and phosphatase inhibitor (KeyGene Biotech, China). Proteins (30 μg) were segregated by sodium dodecyl sulfate–polyacrylamide gel electrophoresis (SDS–PAGE) and then electrophoretically transferred the protein onto a polyvinylidene difluoride (PVDF) membrane. After blocking with 5% bovine serum albumin (BSA) for 2 h, the PVDF membranes were incubated with specific primary antibodies in recommended dilution ratio at 4°C overnight. The primary antibodies used in this study included anti-glyceraldehyde-3-phosphate dehydrogenase (GAPDH) antibody (1:5,000, 60004-1-lg, Proteintech, China), anti-DGCR8 antibody (1:1,000, 10996-1-AP, Proteintech, China), and rabbit anti-RUNX3 antibody (1:1,000, ab135248, Abcam). Subsequently, the PVDF membranes were incubated with secondary antibodies (BOSTER, China) for 2 h. The protein strips were visualized and detected using a chemiluminescence reagent [enhanced chemiluminescence (ECL)] kit (Beyotime, China).

### Quantitative Real-Time PCR

Total RNA was extracted using TRIzol reagent (Invitrogen, Carlsbad, CA), and the reverse-transcription reactions were using PrimeScript RT Master Mix (Takara, Japan). Real-time PCR was using TB Green Fast qPCR Mix (Takara, Japan) according to the instructions. LineGene 9600 Plus Real Time PCR system (Bioer, China) was adopted, and GAPDH was used as a reference. The 2^−ΔΔCt^ method was used to determine the relative quantitation of gene expression. The primer sequences were listed in Table 1.

**Table 1:** The primer sequences for RT-qPCR.

**Gene**	**Primer**
HPV E7	Forward: CAGAGGAGGAGGATGAAATAGATG Reverse: CACAACCGAAGCGTAGAGTC
DGCR8	Forward: GTGCATGCTTGTCCCTTTGG Reverse: TGCCAACATACCTCGTAGGG
Hsa-miR-106a	Forward: GAAAAGTGCTTACAGTGCAG Reverse: GTCCAGTTTTTTTTTTTTTTTCTACCT
GAPDH	Forward: CGCTCTCTGCTCCTCCTGTTC Reverse: ATCCGTTGACTCCGACCTTCAC
U6	Forward: CTCGCT TCGGCAGCA Reverse: AACGCT TCACGAATT TGC

*GAPDH, glyceraldehyde-3-phosphate dehydrogenase; HPV, human papillomavirus; RT-qPCR, quantitative real-time PCR*.

### Immunofluorescent Staining Assay

Cells plated in 24-well plates were fixed with 4% paraformaldehyde in phosphate buffered saline (PBS) for 10 min, permeabilized in 0.5% Triton X-100 for 20 min, washed twice in PBS, and then blocked with 3% BSA (KeyGene Biotech, China) in PBS. After 1 h, the cells were incubated with anti-DGCR8 antibody (1:500, 10996-1-AP, Proteintech, China) at 4°C overnight, then with Alexa Fluor 488 secondary antibody (1:200, SA00013-2, Proteintech, China) for 1 h at 37°C. The nuclei were stained with 5 μg/ml 4′,6-diamidino-2-phenylindole (DAPI) (KeyGene Biotech, China) for 5 min and viewed with a fluorescence microscope (Olympus, Japan).

### Colony Formation Assay

Cells were seeded in 6-well plates and cultured for 14 days upon 4 Gy radiation. The cells were fixed using cold methanol for 20 min and stained with 0.1% crystal violet for 10 min. The number of colonies was used to evaluate the clone formation ability.

### Dual Luciferase Reporter Gene Assay

The wide type (WT) according to the binding site between hsa-miR-106a and RUNX3 and the mutant type (MUT) were, respectively, inserted into the psiCHECK2 (Promega, USA). Subsequently, WT or MUT reporter vectors were co-transfected with hsa-miR-106s mimics or inhibitor. The luciferase activity was measured using dual luciferase reporter gene assay kit (Beyotime, China). Renilla luciferase was used as an internal reference.

### Statistical Analysis

SPSS 21.0 (IBM, United States) was adopted for statistical analysis. Data were shown as mean ± standard derivation (mean ± SD). Student *t*-test was used to analyze differences between two groups, and the *p*-value was acquired from two-tailed tests. A *p* < 0.05 was considered statistically significant. All experiments were repeated in triplicate.

## Results

### Human Papillomavirus E7 Upregulates DGCR8 Expression in Head and Neck Squamous Cell Carcinoma Cells

**Figure 1:**
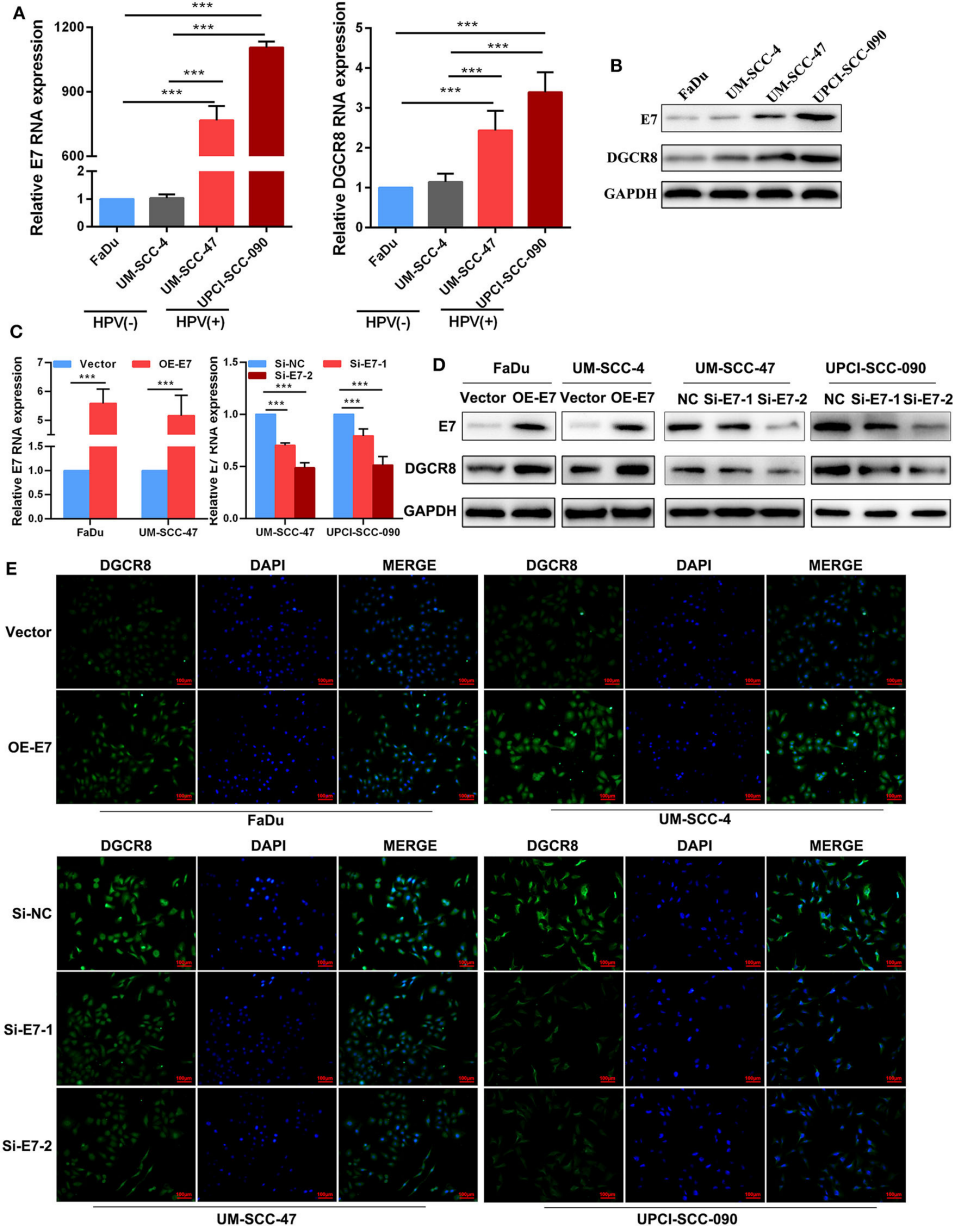
Human papillomavirus (HPV) E7 upregulates DGCR8 expression in head and neck squamous cell carcinoma (HNSCC) cells. **(A)** The mRNA expressions of HPV E7 and DGCR8 in FaDu, UM-SCC-4, UM-SCC-47, and UPCI-SCC-090. **(B)** The protein expressions of HPV E7 and DGCR8 in FaDu, UM-SCC-4, UM-SCC-47, and UPCI-SCC-090. **(C)** The mRNA expression of HPV E7 in FaDu and UM-SCC-4 with E7 overexpression and UM-SCC-47 and UPCI-SCC-090 with E7 silencing. **(D)** The protein expressions of HPV E7 and DGCR8 in FaDu and UM-SCC-4 with E7 overexpression and UM-SCC-47 and UPCI-SCC-090 with E7 silencing. **(E)** HPV E7 upregulated DGCR8 expression in HPV-negative/positive cell lines by immunofluorescence. ***p < 0.001.

According to previous studies, we had proved that FaDu and UM-SCC-4 were HPV-negative cell lines and do not express HPV E7 proteins, while UM-SCC-090 and UM-SCC-47 were HPV-positive cell lines expressing E7 proteins (22). The mRNA (Figure 1A) and protein (Figure 1B) expressions of E7 in HPV-positive cell lines were higher than those of HPV-negative cell lines. Besides, the content of DGCR8 was also much higher in HPV-positive cell lines than that in HPV-negative cell lines (Figures 1A,B). Previous studies had revealed that DGCR8 expression was related to E7. The expression of DGCR8 was measured in HPV-negative cell lines with E7 overexpression and HPV-positive cell lines with E7 silencing. As shown in Figures 1C,D, DGCR8 expression was downregulated as E7 silencing, while the opposite result was obtained after E7 overexpression. We confirmed the above results by using immunofluorescence analysis (Figure 1E). Results presented above suggested that HPV E7 upregulated DGCR8 expression in HNSCC cells.

### DGCR8 Promotes Hsa-miR-106a Transcription and Enhances Radiation Sensitivity

**Figure 2:**
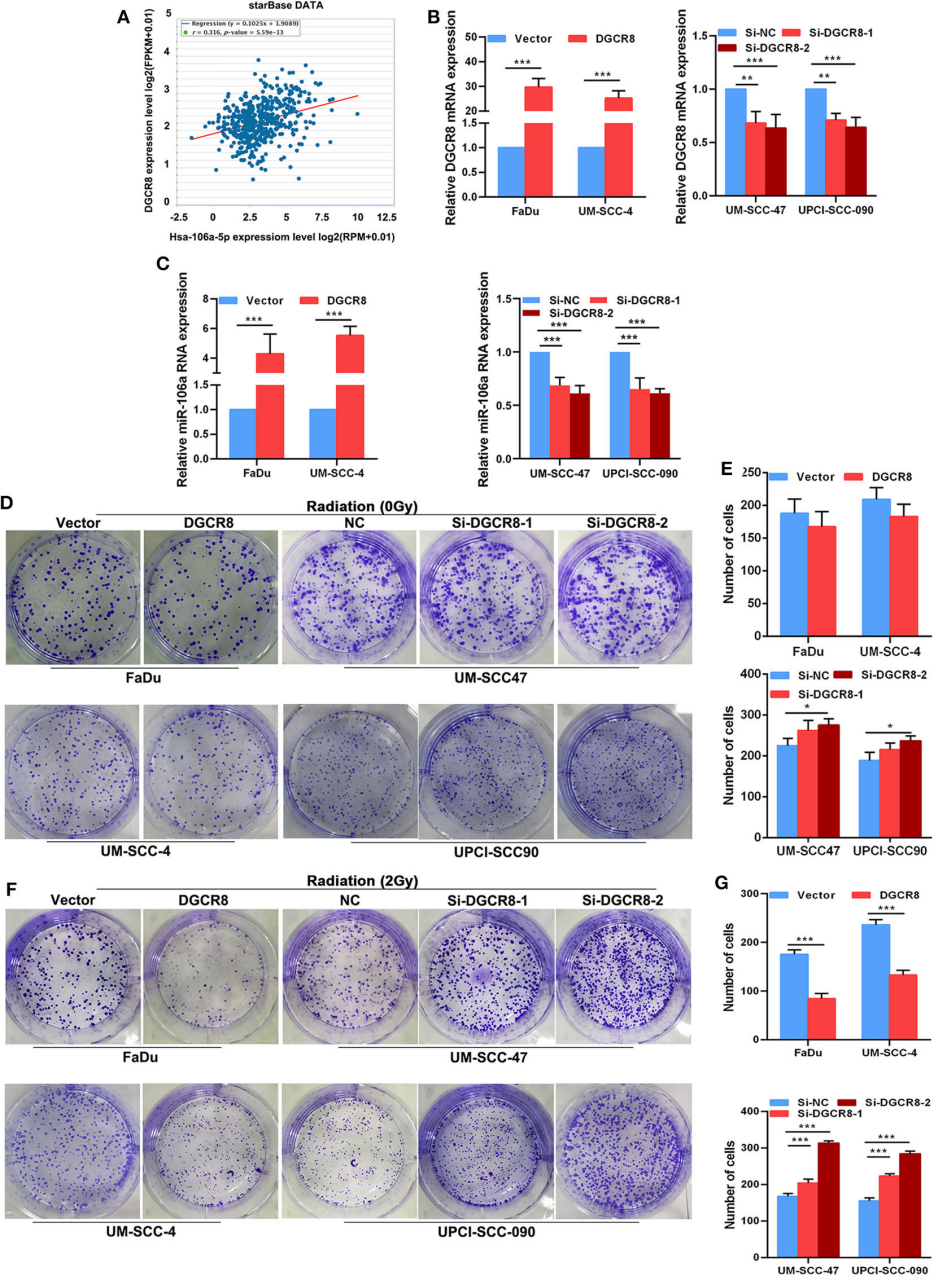
DGCR8 promotes hsa-miR-106a transcription and enhances radiation sensitivity. **(A)** Correlation between DGCR8 and hsa-miR-106a based on TCGA-HNSC cohort. **(B)** The mRNA expression of DGCR8 in the human papillomavirus (HPV)-positive and HPV-negative cells. **(C)** Expression level of hsa-miR-106a was measured in HPV-negative cells with DGCR8 overexpression and HPV-positive cells with DGCR8 silencing. **(D,E)** The effect of DGCR8 on cell proliferation was detected by clonogenic survival upon DGCR8 overexpression in HPV-negative cells and DGCR8 silencing in HPV-positive cells. **(F,G)** The sensitivity of the cells to radiation was detected by clonogenic survival upon DGCR8 overexpression in HPV-negative cells and DGCR8 silencing in HPV-positive cells. *p < 0.05, **p < 0.01, ***p < 0.001.

The correlation between DGCR8 and hsa-miR-106a was analyzed according to HNSCC from The Cancer Genome Atlas (TCGA) database. A strong positive correlation was apparent between DGCR8 and hsa-miR-106a (Figure 2A). We had known that the content of DGCR8 was less in HPV-negative cell lines than that in HPV-positive cell lines (Figures 1A,B). RT-qPCR was adopted to measure the expression of DGCR8 after transfection pcDNA3.1-DGCR8 in HPV-negative cells and siRNA-DGCR8 in HPV-positive cells (Figure 2B). DGCR8 overexpression promoted the transcription of hsa-miR-106a in FaDu and UM-SCC-4, while DGCR8 silencing obtained an opposite trend in UM-SCC-4 and UM-SCC-090 (Figure 2C). To investigate the effect of DGCR8 on radiation sensitivity, clone formation assay was used to evaluate the sensitivity of radiation after DGCR8 overexpression in HPV-negative cells and silencing in HPV-positive cells upon radiation. As shown in Figures 2F,G, high expression of DGCR8 increased the sensitivity of cells to radiation and low expression of DGCR8 decreased the sensitivity of cells to radiation. However, DGCR8 rarely affected cell proliferation in non-radiation conditions (Figures 2D,E). Based on the study results described above, DGCR8 promoted hsa-miR-106a transcription and enhanced radiation sensitivity in HNSCC cells.

### Hsa-miR-106a Inhibits the Expression of RUNX3 and Enhances Radiation Sensitivity

**Figure 3:**
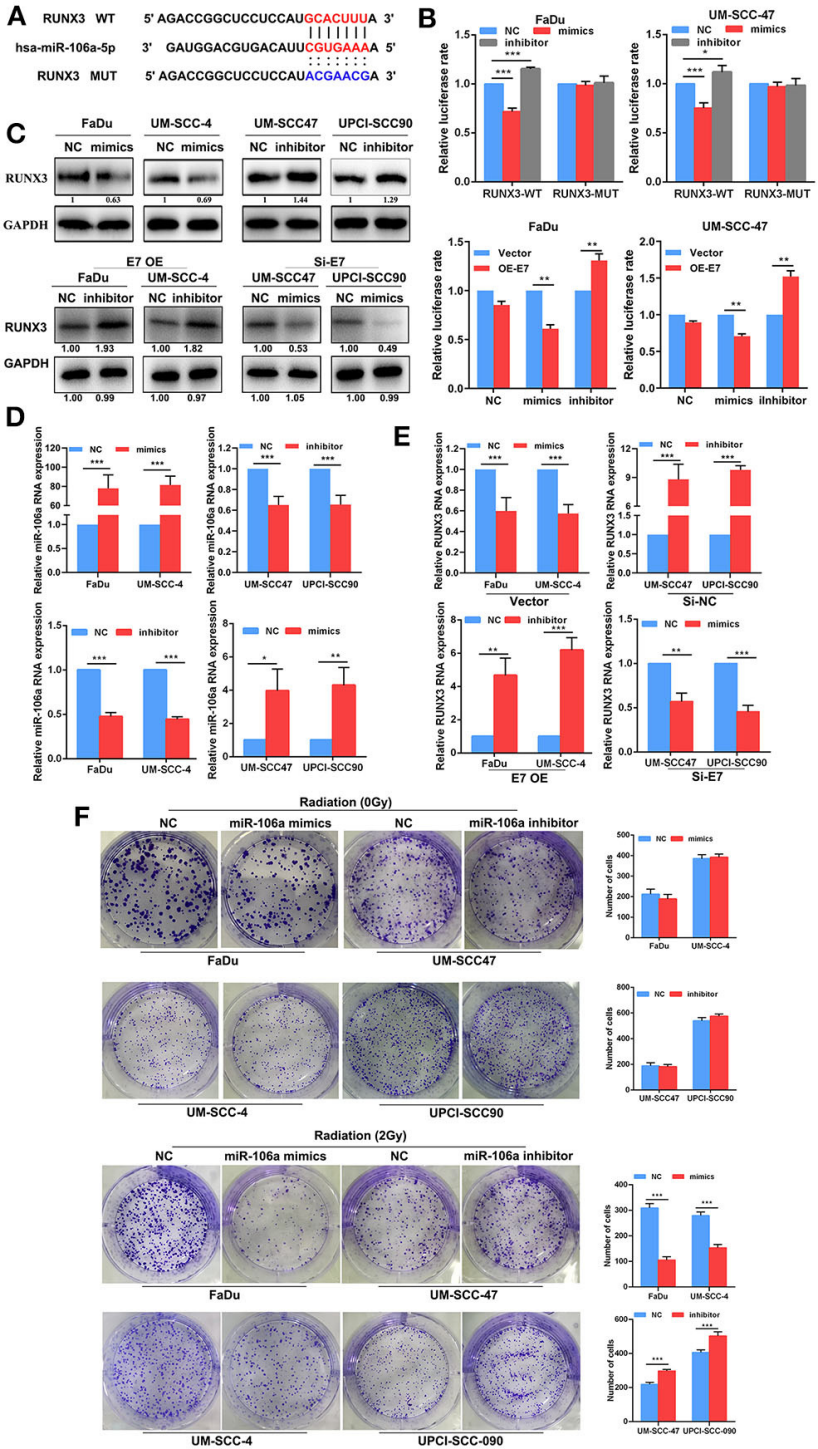
Hsa-miR-106a inhibits the expression of RUNX3 and enhances radiation sensitivity. **(A)** Alignment of the predicted miRNA binding sites in the 3′ untranslated region (UTR) of the RUNX3 mRNA. **(B)** Luciferase reporter gene demonstrated that hsa-miR-106a directly targeted the 3′ UTR of RUNX3. **(C)** The protein expression of RUNX3 in FaDu, UM-SCCC-4, UM-SCC47, and UPCI-SCC90. **(D)** Hsa-miR-106a transfection efficiency was measured by RT-qPCR. **(E)** The mRNA expression of RUNX3 in human papillomavirus (HPV)-negative cells with hsa-miR-106a overexpression and HPV-positive cells with hsa-miR-106a silencing. **(F)** The sensitivity of the cells to radiation was detected by clonogenic survival with the condition of hsa-miR-106a overexpression in HPV-negative cells and hsa-miR-106a silencing in HPV-positive cells. *p < 0.05, **p < 0.01, ***p < 0.001.

miRNAs cannot directly involve protein translation, and its effect on radiation sensitivity must be targeted to regulate mRNA transcription. It has been reported that RUNX3 is involved in the process of DNA damage repair (19, 20), and one of the most important factors affecting radiation sensitivity is the repair of DNA damage. In order to further explore whether hsa-miR-106a is one of the key steps for DGCR8 to promote radiation sensitivity, we found that hsa-miR-106a targeted and regulated RUNX3, which was the key gene of DNA damage repaired by bioinformatics analysis (Figure 3A). The transfection efficiency was as expected in FaDu, UM-SCC-4, UM-SCC-47, and UPCI-SCC-090 (Figure 3D). PsiCHECK2, a luciferase reporter vector, was constructed with the 3′ untranslated region (UTR) sequence of RUNX3 and co-transfected mimics or inhibitor of hsa-miR-106a to explore the regulation of hsa-miR-106a on RUNX3. As shown in Figure 3B, hsa-miR-106a overexpression downregulated the relative luciferase rate, while hsa-miR-106a silencing upregulated the relative luciferase rate in Fadu and UM-SCC-47 with RUNX3-WT plasmid co-transfection. The relative luciferase rate was rarely changed in the RUNX3-MUT group. E7 overexpression downregulated the relative luciferase rate compared with vector group in FaDu and UM-SCC-47 cells (Figure 3B). It indicated that hsa-miR-106a could inhibit the transcription of RUNX3 by targeting its 3′ UTR and HPV E7 affected the regulation of hsa-miR-106a on RUNX3. In addition, it could negatively regulate the gene expression in the mRNA and protein levels of RUNX3 (Figures 3C,E). A clone formation assay was performed to investigate the impact of hsa-miR-106a on radiation sensitivity in HNSCC cells. In 2Gy radiation condition, the number of clone formation in hsa-miR-106a overexpression group was much more than that of the negative control (NC) group in FaDu and UM-SCC-4; however, the number of clone formation in the inhibitor group was less than that of the NC group in UM-SCC-47 and UPCI-SCC-090 (Figure 3F). However, miR-106a rarely increased the clone formation number in non-radiation conditions. The results above suggested that hsa-miR-106a inhibited the transcription of RUNX3 by targeting its 3′ UTR and enhanced radiation sensitivity.

### Human Papillomavirus E7/DGCR8 Inhibits the Expression of RUNX3 and Affects Radiation Sensitivity by Promoting the Expression of hsa-miR-106a

**Figure 4:**
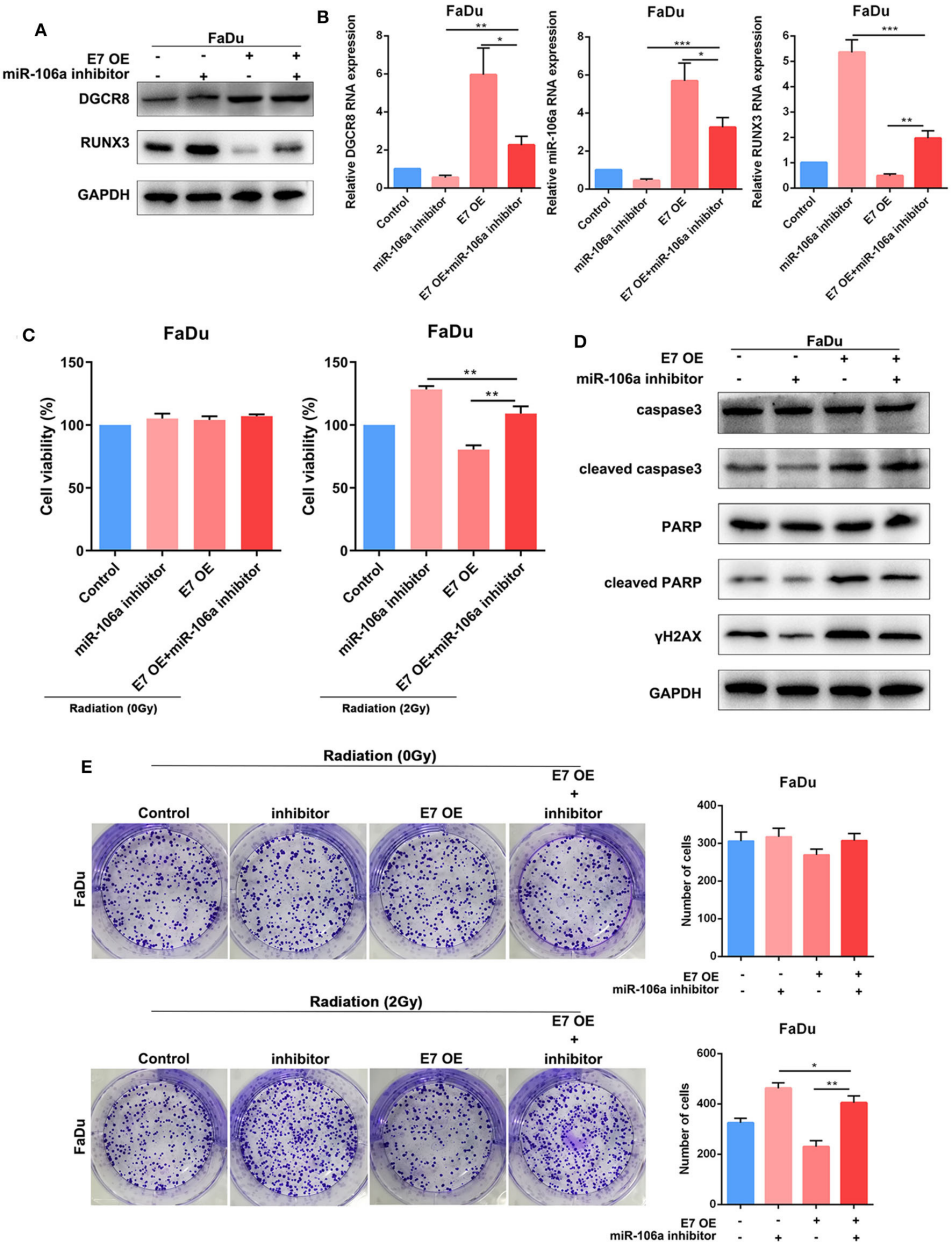
DGCR8 inhibits the expression of RUNX3 and affects radiation sensitivity by promoting the expression of hsa-miR-106a. **(A)** Western blot showing rescue of RUNX3 expression level in FaDu. **(B)** Quantitative real-time PCR (RT-qPCR) was performed to detect DGCR8, miR-106a, and RUNX3 expression level in FaDu. **(C)** Cell Counting Kit-8 (CCK-8) showing rescue of cell viability in FaDu in radiation or non-radiation condition. **(D)** The clone formation assay showing rescue of radiation sensitivity in FaDu. **(E)** The protein expressions of apoptosis-related caspase-3, PARP, and DNA damage-related γH2AX were measured by Western blot. *p < 0.05, **p < 0.01, ***p < 0.001.

Previously, we had confirmed the relationship in DGCR8/hsa-miR-106a and hsa-miR-106a/RUNX3, as well as the effects on radiation or non-radiation sensitivity. Here, we assumed that HPV E7/DGCR8 inhibited the expression of RUNX3 and enhanced radiation sensitivity by promoting the expression of hsa-miR-106a. Rescue experiments were performed to examine whether radiation sensitivity promoted by HPV E7/DGCR8 were achieved by downregulation of hsa-miR-106a. As shown in Figures 4A,B, the content of RUNX3 decreased after DGCR8 overexpression, while the expression of RUNX3 increased with hsa-miR-106a inhibitor transfection. In addition, the expression of RUNX3 was rescued after hsa-miR-106 silencing upon DGCR8 overexpression in FaDu (Figures 4A,B). The clone formation assay was used to measure sensitivity on radiation. The number of clone formation was increased after hsa-miR-106a rescue upon radiation, while the number of clone formation changed little in non-radiation condition, indicating has-miR-106a could increase radiosensitivity (Figure 4D). Cell Counting Kit-8 (CCK-8) assay was used to measure cell viability. The results indicated that cell viability increased after hsa-miR-106a rescue upon radiation and supported the results presented above (Figure 4C). The protein expressions of apoptosis-related caspase-3, poly(ADP-ribose) polymerase (PARP), and DNA damage-related γH2AX were measured by Western blot. Cleaved caspase-3, cleaved PARP, and γH2AX decreased after hsa-miR-106a silencing upon DGCR8 overexpression (Figure 4E). It indicated that hsa-miR-106a promoted DNA damage and apoptosis induced by radiation in FaDu under radiation condition. However, neither DGCR8 nor miR-106a had effects on DNA damage and apoptosis caused by radiation. The above experiments indicated that DGCR8 inhibited the expression of RUNX3 and affected radiation sensitivity by promoting the expression of hsa-miR-106a.

**Figure 5:**
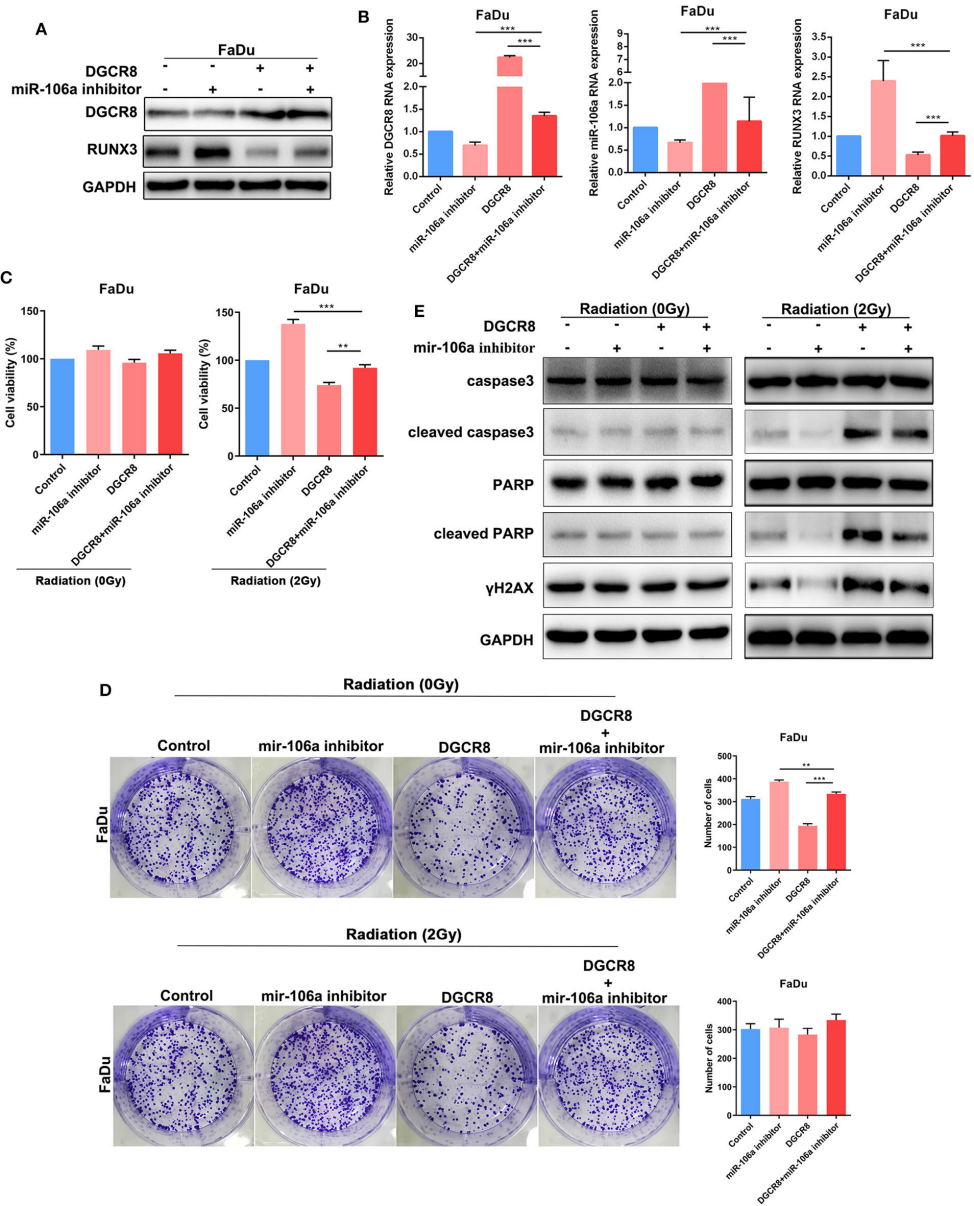
Human papillomavirus (HPV) E7 inhibits the expression of RUNX3 and affects radiation sensitivity by promoting the expression of hsa-miR-106a. **(A)** Western blot showing rescue of RUNX3 expression level in FaDu. **(B)** Quantitative real-time PCR (RT-qPCR) was performed to detect DGCR8, miR-106a, and RUNX3 expression levels in FaDu. **(C)** Cell Counting Kit-8 (CCK-8) showing rescue of cell viability in FaDu in radiation or non-radiation condition. **(D)** The protein expressions of apoptosis-related caspase-3, PARP, and DNA damage-related γH2AX were measured by Western blot. **(E)** The clone formation assay showing rescue of radiation sensitivity in FaDu. **p < 0.01, ***p < 0.001.

In Figures 5A,B, E7 overexpression evaluated DGCR8 expression in FaDu and decreased RUNX3 content. Knockdown of miR-106a promoted RUNX3 expression in FaDu. The clone formation assay was used to measure the content of E7 and miR-106a on radiosensitivity. The number of clone formation was increased after miR-106a knockdown upon radiation, while the number of clone formation rarely changed in non-radiation condition, indicating has-miR-106a could increase radiosensitivity (Figure 5E). HPV E7 and miR-106a could not affect cell viability in non-radiation condition. However, knockdown of miR-106a increased cell viability, while E7 overexpression decreased it in radiation condition (Figure 5C). Cleaved caspase-3, cleaved PARP, and γH2AX decreased after hsa-miR-106a silencing upon E7 overexpression (Figure 5D). It indicated that HPV E7 inhibited the expression of RUNX3 and affected radiation sensitivity by promoting the expression of hsa-miR-106a.

### Diagram of the Human Papillomavirus E7/DGCR8/hsa-miR-106a Axis Enhances Radiation Sensitivity

**Figure 6:**
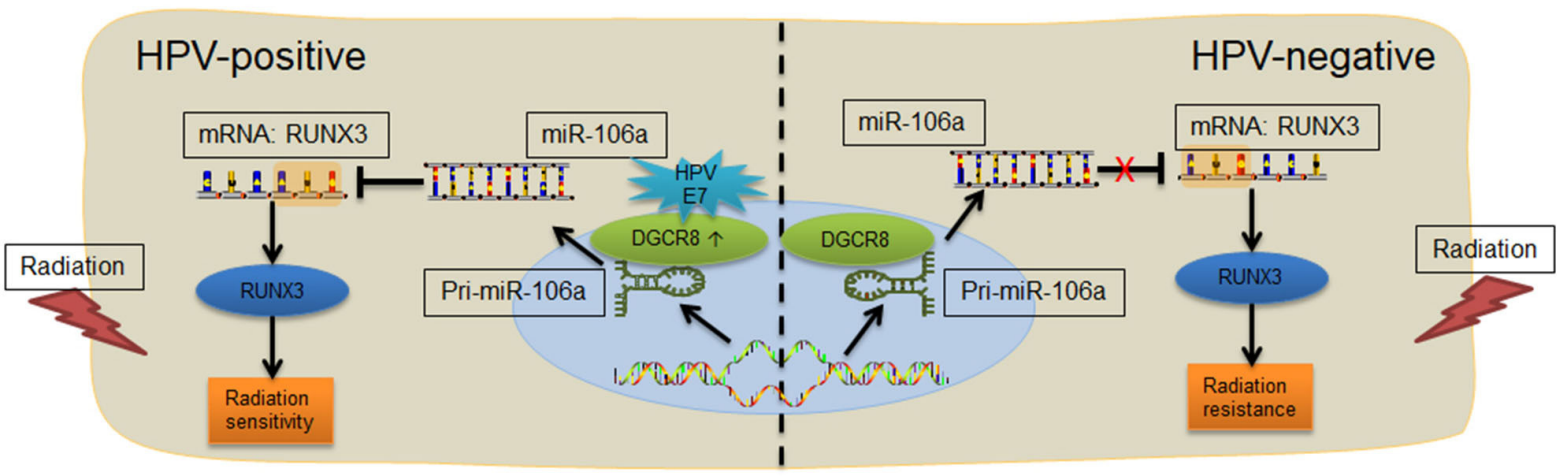
Diagram of the E7/DGCR8/hsa-miR-106a axis enhances radiation sensitivity. Human papillomavirus (HPV)-positive head and neck squamous cell carcinoma (HNSCC) with high DGCR8 expression promoted the expression of miR-106a. The miR-106a suppressed RUNX3 mRNA expression *via* binding to the 3′ untranslated region (UTR) of RUNX3 and enhanced radiation sensitivity.

According to previous studies, we have known that HPV-positive HNSCC is more sensitive to radiation. Through the detection of HNSCC tumor tissue and the analysis of TCGA database, we found that DGCR8, a protein that affected the maturation of miRNA, promoted the transcription of hsa-miR-106a and had a strong correlation. In addition, hsa-miR-106a targeted binding to 3′ UTR region of RUNX3, a key protein for DNA damage repair and inhibited its transcription (Figure 6). In an overview, HPV E7/DGCR8/hsa-miR-106a axis enhanced radiation sensitivity. It is expected to be an effective treatment for radiation-insensitive HNSCC patients by targeting DGCR8 or hsa-miR-106a.

## Discussion

Previous studies have already shown that the radiation sensitivity of HNSCC is closely related to HPV E7. To further explore the underlying mechanism between E7 expression and radiation sensitivity, we found that DGCR8, a protein impacting the process of miRNA maturation, promoted hsa-miR-106a transcription in HNSCC cell lines. Besides, hsa-miR-106a targeted binding to 3′ UTR region of RUNX3 to inhibit its transcription and radiation resistance in HNSCC. In conclusion, HPV E7/DGCR8/hsa-miR-106a axis enhanced radiation sensitivity in HNSCC.

HPV is a major agent of HNSCC according to epidemiological data (23). In the present meta-analysis, the average incidence rate of HPV-associated HNSCC is 42.62% (24). Lajer et al. (25) found that HPV-positive HNSCC had a distinct miRNA profile compared with HPV-negative HNSCC. DGCR8 regulates multiple signaling pathways through miRNA-regulated genes (26). In our study, we found that the content of DGCR8 in HPV-positive HNSCC cells was much higher than that of HPV-negative cells (Figure 1). Besides, there was a strong correlation between DGCR8 and hsa-miR-106a (Figure 2). As we have known in previous studies (22), HPV-positive HNSCC cells were highly sensitive to radiation, and HPV-positive cells were rich in DGCR8 and hsa-miR-106a. Ardenne and Reitnauer (27) demonstrated that hsa-miR-106a suppressed proliferation and induced apoptosis. Application of miR-106a mimics induced cerebrovascular endothelial cell death under oxygen-glucose deprivation conditions (28). Therefore, DGCR8 promoting hsa-miR-106a transcription might be one of the important reasons for enhancing radiation sensitivity.

Based on current research, miRNAs are deemed to regulate more than 60% of human protein coding genes (29). It does not participate in protein translation but affects the content of many proteins. Non-small-cell lung cancer patients with a high RUNX3 level exhibited a significantly higher apoptosis index than that with a low level of RUNX3 (30). It participated in cell growth and apoptosis. Exogenous RUNX3 expression decreased cell proliferation and increased gemcitabine sensitivity in endogenous RUNX3-negative cell lines (31). In our study, we demonstrated that the expression of RUNX3 was negatively regulated by has-miR-106a in HNSCC cell lines (Figures 3A–C). In addition, RUNX3 silencing increased the sensitivity of HNSCC cells to radiation (Figure 3F). It was a key suppressor protein regulated by HPV E7/DGCR8/miR-106a axis to increase radiation sensitivity in HNSCC. Therefore, blocked RUNX3 might enhance the efficacy of radiation and reverse radioresistance in HNSCC patients.

Overall, the expression of DGCR8 in HPV-negative cells was lower than that in HPV-positive cells in HNSCC cells. It inhibited has-miR-106a transcription and disinhibition of RUNX3 expression. RUNX3 was a key protein to cause radioresistance. This might be one of the important reasons why HPV-negative HNSCC was not sensitive to radiotherapy. We can target to increase the content of has-miR-106a or reduce the content of RUNX3 to improve radiotherapy sensitivity of radioresistant patients.

## Conclusions

Our study indicated that HPV E7 activated DGCR8/miR-106a/RUNX3 axis to enhance radiosensitivity. It may enhance the efficacy of radiation and reverse radioresistance in HNSCC patients and provides directions for targeted therapeutic interventions.

## Data Availability Statement

Publicly available datasets were analyzed in this study. This data can be found at: https://www.cancer.gov/about-nci/organization/ccg/research/structural-genomics/tcga.

## Author Contributions

ZL and CZ designed the study. All authors contributed to data analysis, drafting and revising the article, gave final approval of the version to be published, and agree to be accountable for all aspects of the work.

## Conflict of Interest

The authors declare that the research was conducted in the absence of any commercial or financial relationships that could be construed as a potential conflict of interest.

## Abbreviations

HPV: Human Papillomavirus
DGCR8: Microprocessor complex subunit DGCR8
RUNX3: Runt-related transcription factor 3
PARP: Poly (ADP-Ribose) Polymerase 1
CCK-8: Cell Counting Kit-8
UTR: Untranslated region
pRb: Retinoblastoma protein
ATCC: American Type Culture Collection
DMEM: Dulbecco's Modified Eagle's Medium
MEM: Minimum Essential Medium
RIPA: Radio Immunoprecipitation Assay
PMSF: Phenylmethanesulfonyl fluoride
SDS-PAGE: Sodium dodecyl sulfate polyacrylamide gel electrophoresis
BSA: Bovine Serum Albumin
GAPDH: Glyceraldehyde-3-Phosphate Dehydrogenase
ECL: Electrochemiluminescence
PBS: Phosphate buffer saline
DAPI: 4′,6-diamidino-2-phenylindole
TCGA: The Cancer Genome Atlas.
